# mTORC2 Phosphorylation of Akt1: A Possible Mechanism for Hydrogen Sulfide-Induced Cardioprotection

**DOI:** 10.1371/journal.pone.0099665

**Published:** 2014-06-20

**Authors:** Yue Zhou, Daying Wang, Xiufang Gao, Karsheng Lew, Arthur Mark Richards, Peipei Wang

**Affiliations:** 1 Cardiovascular Research Institute, National University of Singapore, Singapore, Singapore; 2 Department of Cardiology, Putuo Hospital, Shanghai, China; 3 Department of Cardiology, Huashan Hospital, Fudan University, Shanghai, China; Virginia Commonwealth University, United States of America

## Abstract

Hydrogen sulfide (H_2_S) is known to have cardiac protective effects through Akt activation. Akt acts as a ‘central sensor’ for myocyte survival or death; its activity is regulated by multiple kinases including PI3K, mTORC2, PDK1 and phosphatases including PTEN, PP2A and PHLPPL. Based on the previous finding that PI3K inhibitor LY294002 abolishes H_2_S-induced Akt phosphorylation and cardioprotection, it is accepted that PI3K is the mediator of H_2_S-induced Akt phosphorylation. However, LY294002 inhibits both PI3K and mTOR, and PI3K only recruits Akt to the membrane where Akt is phosphorylated by Akt kinases. We undertook a series of experiments to further evaluate the role of mTORC2, PDK1, PTEN, PP2A and PHLPPL in H_2_S-induced Akt phosphorylation and cardioprotection, which, we believe, has not been investigated before. Hearts from adult Sprague-Dawley rats were isolated and subjected to (i) normoxia, (ii) global ischemia and (iii) ischemia/reperfusion in the presence or absence of 50 µM of H_2_S donor NaHS. Cardiac mechanical function and lactate dehydrogenase (LDH) release were assessed. All hearts also were Western analyzed at the end of perfusion for Akt and a panel of appropriate Akt regulators and targets. Hearts pretreated with 50 µM NaHS had improved function at the end of reperfusion (Rate pressure product; 19±4×10^3^ vs. 10±3×10^3^ mmHg/min, p<0.05) and reduced cell injury (LDH release 19±10 vs. 170±87 mU/ml p<0.05) compared to untreated hearts. NaHS significantly increased phospho-Akt, phospho-mTOR, phospho-Bim and Bcl-2 in reperfused hearts (P<0.05). Furthermore using H9c2 cells we demonstrate that NaHS pretreatment reduces apoptosis following hypoxia/re-oxygenation. Importantly, PP242, a specific mTOR inhibitor, abolished both cardioprotection and protein phosphorylation in isolated heart and reduced apoptotic effects in H9c2 cells. Treating hearts with NaHS only during reperfusion produced less cardioprotection through a similar mechanism. These data suggest mTORC2 phosphorylation of Akt is a key mediator of H_2_S-induced cardioprotection in I/R.

## Introduction

Hydrogen sulfide (H_2_S) was first identified in 1996 [1] as an important endogenous regulator of a wide range of cell functions [2], [3], [4], [5]. In the cardiovascular system H_2_S produces three important effects. First, it induces the relaxation of isolated blood vessels [4] and serves as an *in vivo* regulator of blood pressure [2], [5]. Second, it has negative chronotropic and inotropic effects on heart muscle [3]. Third, H_2_S potently protects against ischemia/reperfusion (I/R) injury in myocytes, in isolated hearts and in intact animals [6], [7], [8], [9], [10]. The activation of myocardial Akt is an important mediator of this ischemic cardioprotection [11], [12], [13], [14]. However, all potential molecular mechanisms underpinning H_2_S-related cardioprotective Akt activation is not fully known.

Phosphorylation and de-phosphorylation of Akt-Ser473 and Akt-Thr308 regulates the activity of this kinase. Since the phospho-inositide-3-kinase (PI3K) signaling pathway is believed to result in the phosphorylation of these two residues, early studies focused on the role of PI3K in H_2_S cardioprotection. Indeed, the putative PI3K inhibitor LY294002 reduces H_2_S-induced Akt phosphorylation and cardioprotection [7], [15]. However, LY294002 inhibits not only PI3K but also mammalian target of rapamycin (mTOR) and other protein kinases [16], [17]. In addition, PI3K does not directly activate Akt. Indeed, binding of PIP_3_, the down-stream product of PI3K, to Akt recruits Akt to membranes where it is subsequently phosphorylated by other kinases [18]. As mTORC2 also phosphorylates Akt [19], it may be an unrecognized contributor to H_2_S cardioprotection. Other potential modulators of Akt activity include (i) the tyrosine phosphatase Phosphatase and Tensin homolog (PTEN) which regulate Akt activity through dephosphorylation of phosphoinositide PIP_3_ down-stream of PI3K [20], (ii) 3-phosphoinositide dependent protein kinase-1 (PDK1) [21], and (iii) PH domain and leucine rich repeat protein phosphatases 2 (PHLPPL or PHLPP2) and protein phosphatase 2 (PP2A) which dephosphorylate and inhibit Akt [22], [23]. All of these regulators except PI3K have not been investigated in H_2_S-induced Akt phosphorylation in the heart.

While Akt activation is critical for ischemic cardioprotection, the downstream targets for Akt in this setting remain unresolved. Increasing experimental evidence shows that the Bcl-2 family is a critical mediator of cardiac ischemia/reperfusion injury through activation of myocyte apoptotic signaling [24], [25]. It is not clear whether Akt activated by H_2_S during ischemia/reperfusion might regulate Bcl-2 and Bim which would decrease apoptosis and thereby contribute to cardioprotection.

Thus this study had two purposes. First we investigated whether up-stream regulators other than PI3K can regulate Akt during H_2_S-cardioprotection. Second we sought to identify potential Akt down-stream effectors which protect hearts against ischemic/reperfusion. Our data demonstrate that mTORC2 can activate Akt in ischemic hearts treated with H_2_S, and that inhibition of Bim signaling coupled with an increase in Bcl-2 may be intrinsic to the molecular mechanisms of H_2_S cardioprotection.

## Materials and Methods

This study was approved by the Institutional Animal Care and Use Committee of National University of Singapore and complied with the Guide for the Care and Use of Laboratory Animals published by the National Institutes of Health (NIH Publication No. 85-23, Revised 1996). Sixty-five male Sprague-Dawley rats (250–350 g) were used in this study. All rats were kept in a temperature-controlled room (21±2°C) with 12 hours light and dark cycle. Water and diet were available ad libitum. All perfusions were performed during the light cycle without fasting.

All chemicals were purchased from Sigma-Aldrich (Sigma-Aldrich Co, LLC, Singapore) unless stated otherwise. NaHS was used as a H_2_S donor as it readily enters aqueous solutions and releases H_2_S, Na^+^, HS^−^ and H^+^. At pH 7.4 approximately 18.5% of the sulfide exists as H_2_S [26]. The reported tissue concentrations of H_2_S vary from a less than 10 up to 100 µM depending on the method of measurement [26]. The mouse left ventricle produces approximately 15 nmol H_2_S/g/min and 10 µM H_2_S provokes vasodilatation in vitro [4]. Here we used 50 µM NaHS as an H_2_S donor as this dose should generate ∼10 µM H_2_S. PP242, a specific mTOR inhibitor, was dissolved in DMSO as a 0.1 mM stock which was diluted in the perfusion buffer to achieve a final concentration of 0.3 µM.

### Isolated heart preparation

The methods for the isolated heart preparation and Langendorff perfusion have been detailed previously [27]. All Langendorff-perfused hearts beat in sinus rhythm. A fluid-filled balloon inserted into the left ventricle was used to set the end-diastolic pressure to between 0 and 5 mmHg. Hearts were perfused at 75 mmHg constant pressure by adjusted coronary artery flow rate. Cardiac function was recorded throughout perfusion (LabChart Pro, Advance Tech Pte Ltd, Singapore).

### Perfusion protocols

**Figure 1:**
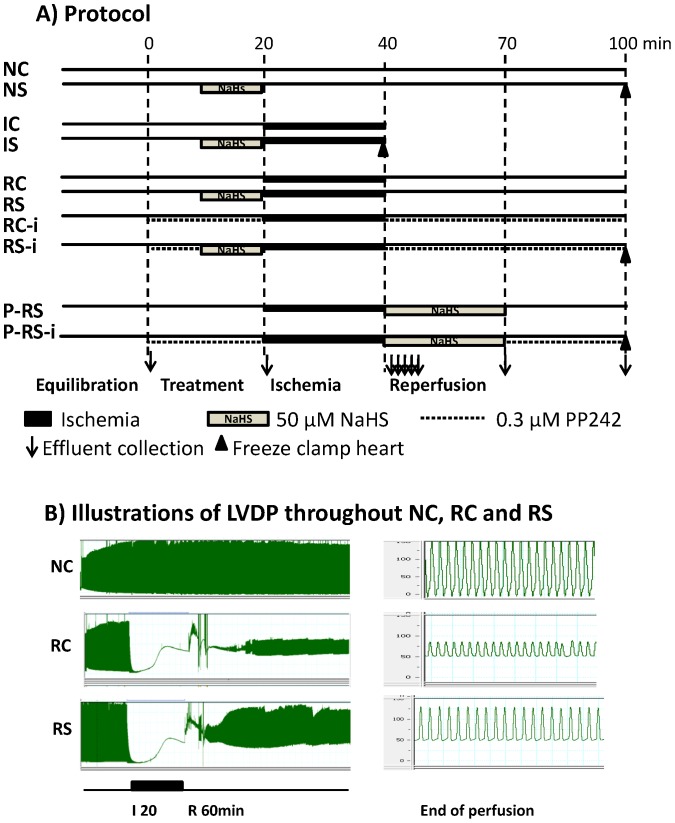
Perfusion protocol and an illustration of LVDP. (A) Protocol: There are total of 10 groups of hearts perfused under three different conditions: normoxia (N), 20 min global zero flow ischemia (I) and I followed by 60 min reperfusion (R). Each perfusion condition was further divided into control (C), 50 µM NaHS, a H_2_S donor, pretreatment for 10 min (S) or post-treatment for 30 min (P–S) with or without 0.3 µM PP242, a specific mTORC2 inhibitor (-i). The 10 groups studied are NC, NS, IC, IS, RC, RS, RC-i, RS-i, P-RS and P-RS-i; n = 5 to 10 each group. (B) Illustrations of LVDP throughout perfusions under Normoxia, I/R control and I/R NaHS pretreatment (NC, RC, RS).

After 10 to 20 min of equilibration of normoxic perfusion, hearts were divided into the following 10 groups (n = 5 to10 heart per group) in this study (Fig. 1A). (1) Hearts perfused normoxically for 60 min (Group NC); (2) Hearts subjected to global zero flow ischemia (I) for 20 min (Group IC); (3) Hearts subjected to I followed by 60 min reperfusion (Group RC); (4) Hearts treated with 50 µM NaHS for 10 min followed by 60 min normoxic perfusion (Group NS); (5) Hearts pre-treated with 50 µM NaHS for 10 min subjected to I (Group IS); (6) Hearts pre-treated with 50 µM NaHS for 10 min subjected to 20 min of I and 60 min of R (Group RS); (7) Hearts subjected to I in the absence of NaHS and then exposed to 50 µM NaHS only during R (Group P-RS); (8) Hearts subjected to I/R in the absence of NaHS that were pre-treated with 0.3 µM of PP242, an mTORC2 specific inhibitor (Group RC-i); (9) Hearts pre-treated with 50 µM NaHS for 10 min in the presence of PP242 then subjected to I/R (Group RS-i); and (10) Hearts subjected to I and then exposed to 50 µM NaHS and PP242 only during R (Group P-RS-i).

Coronary effluent was collected during pretreatment, before ischemia, every minute during the first 5 min of reperfusion, and at 30 and 60 min of reperfusion. At the end of perfusion, hearts were freeze-clamped and stored at −80°C for further analysis.

### Western Blot analyses

Protocols were adapted from our previous work [28]. In brief, frozen myocardium tissue was ground in liquid nitrogen. Then ≅50 mg myocardium tissue powder was lysed in 500 µl lysis buffer containing 1∶100 protease inhibitor cocktail, 2 mM Na_3_VO_4_ and 10 mM NaF. Samples were sonicated (MicrosonTM XL-2000, Qsonica LLC. Newtown, CT, USA) and centrifuged at 14,000 g for 30 min. Supernatant protein concentrations were measured (Bio-Rad Laboratories (S) Pte Ltd, Singapore) and 25 µg of each sample was loaded onto 5 to 14% SDS-PAGE gels and electrophoresed. After over-night transfer at 30 V (4°C), membranes were immunoblotted with antibodies recognizing (i) p-Akt (Ser473), (ii) p-Akt (Thr308), (iii) total Akt, (iv) p-PTEN(Ser380/Thr382/383), (v) total PTEN, (vi) PDK1, (vii) PP2A, (viii) p-Bim (Ser69), (ix) total Bim, (x) Bcl-2, (xi) Bax, (xii) p-mTOR (Ser2448), and (xiii) total mTOR (Cell Signaling Technology, Research Biolabs Pte Ltd, Singapore), and (xiv) PHLPPL (Santa Cruz Biotechnology Inc., TWC BIO Pte Ltd, Singapore). GAPDH (Abcam, Abcell Pte Ltd, Singapore) was used as loading control. The chemiluminescence signal was captured with G:Box Chemi XL 1.4 (Syngene, Insta BioAnalytik Pte Ltd, Singapore), intensity was calculated with ImageJ software (www.imagej.nih.gov) and protein expression was normalized to total Akt, PTEN, Bim, Bcl-2, mTOR or GAPDH.

### Lactate dehydrogenase (LDH) release

Effluent LDH concentration was measured spectrophotometrically (TOX-7, Sigma-Aldrich Co. LLC, Singapore) at 490 nm (EnSpire 2300 Multilabel Reader, PerkinElmer, Singapore). LDH concentration was calculated using an LDH standard curve.

### Assessment of apoptosis and cell death

H9c2, a rat cardiac myoblast cell line, was purchased from ATCC (Manassas, VA, USA). After seeding for 24–48 hr, cells were subjected to normoxia or I/R in which they underwent 15 hr of hypoxia (0.2% O_2_ & serum free) followed by 2 hr of normal incubation (reperfusion). Normoxic and I/R cells were further divided into 4 groups: (i) Control, (ii) 50 µM NaH_2_S, (iii) 0.3 µM PP242, and (iv) NaHS & PP242 combined. At the end of incubation, these 8 groups of cells were trypsinized and apoptosis was assessed (MUSE™ Cell Analyzer, Merck-Millipore, USA). 7-AAD and annexin V double negative, annexin V only positive, annexin V and 7-AAD double positive, and 7-AAD only positive staining indicated cells in status of viable, early apoptosis, late apoptosis, and dead respectively.

### Statistics

All values are presented as mean ± SEM. Data were compared for differences by one-way ANOVA followed by Bonferroni post-hoc analysis or unpaired two tail t test (Graph Pad Prism, San Diego, CA, USA), as appropriate. A p value of less than 0.05 was considered statistically significant.

## Results

Baseline cardiac function measured during a 20 min equilibration did not differ among 10 groups (Table 1). Rate pressure product (RPP = HR×LVDP), an indicator of total cardiac work load, was approximately 40×10^3^ mmHg/min across all groups which reflects well maintained preparations [27], [29].

**Table 1:** Heart weight and baseline cardiac function of all groups in this study.

	n	HW	CF	HR	LVDP	+dP/dt (×10^2^)	−dP/dt (×10^2^)	RPP (×10^3^)
**NC**	6	1.50±0.08	15.0±1.0	280±5	151±5	47±3	28±1	43±0.2
**IC**	6	1.30±0.07*	15.2±1.3	297±14	144±8	46±3	27±2	42±0.3
**RC**	10	1.62±0.04	15.0±0.7	296±8	142±5	48±1	29±1	42±0.2
**NS**	6	1.56±0.10	12.2±1.1	266±11	146±4	53±2	32±1	39±0.2
**IS**	6	1.24±0.03*	12.3±1.9	292±6	138±5	54±2	30±2	40±0.1
**RS**	7	1.75±0.06	16.2±1.1	282±13	138±6	48±3	29±2	39±0.2
**P-RS**	9	1.79±0.06	14.1±0.6	281±19	140±3	47±2	31±1	39±0.2
**RC-i**	5	1.73±0.03	13.2±1.5	305±16	152±12	51±3	33±3	43±0.5
**RS-i**	5	1.72±0.03	14.1±0.6	306±7	142±14	46±2	28±1	43±0.4
**P-RS-i**	5	1.77±0.05	15.8±1.1	308±11	131±6	50±1	31±1	40±.01

Values are means ± SE. Abbreviations used in this table are: HW = heart weight (g); CF = coronary artery flow rate (ml/min); HR = heart rate (bpm); LVDP = left ventricular developing pressure (systolic pressure – end diastolic pressure. mmHg); ±dP/dt (mmHg/sec); RPP = rate pressure products (HR×LVDP, mmHg/min). The representatives for 10 groups are: N = Normoxia, I = Ischemia, R = Reperfusion, C = non-treated Control, S = 50 µM NaHS treatment, i = 0.3 µM PP242, an mTOR inhibitor, and P = post-treatment.

*p<0.05 vs. the rest of groups by one-way ANOVA followed by Bonferroni post-hoc analysis.

### H_2_S increases Akt & mTOR phosphorylation which contributes to cardioprotection

While 100 µM NaHS reportedly protects isolated hearts against reperfusion damage [7], our preliminary analyses found that this dose of NaHS increased coronary flow by 20% and decreased heart function (data not shown). Therefore, we used 50 µM NaHS in our study to avoid this complication. At this concentration coronary flow was increased less than 10% and baseline cardiac function was not significantly changed. Indeed, cardiac function was well preserved with 60 min of normoxic perfusion in the NC and NS groups as the RPP and LVDP were 90% and ±dP/dt was 100% of baseline at the end of perfusions. An illustration of real time recording of LVDP for NC, RC and RS heart is shown in Fig. 1B.

**Figure 2:**
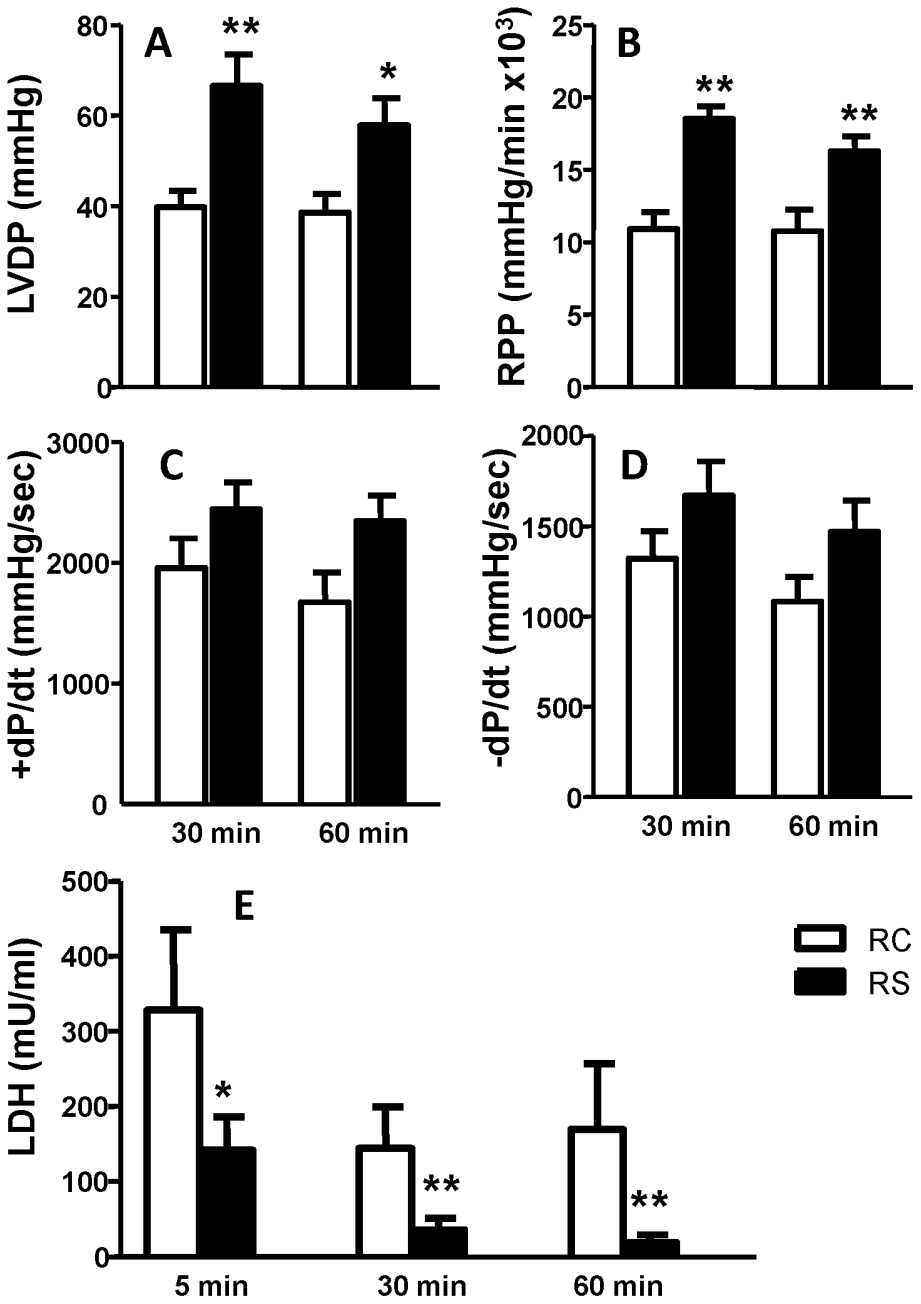
NaHS pretreatment improves cardiac functional recovery and reduces LDH release during reperfusion. (A) LVDP, left ventricular developed pressure; (B) RPP, rate pressure product = LVDP×heart rate; (C) +dP/dt, the rate of increase of pressure over time; (D) −dP/dt, the rate of decrease of pressure over time; (E) LDH release. RC = Reperfusion Control, RS = Reperfusion NaHS pretreatment. *P<0.05, **P<0.01 vs. Reperfusion control (RC) by two-tail unpaired t test.

Pre-treating hearts with 50 µM NaHS afforded cardioprotection as indicated by a significant improvement in LVDP and RPP at 30 and 60 min of reperfusion (Fig. 2A and B). While NaHS also improved +/−dP/dt during reperfusion, this change did not reach statistical significance (Fig. 2C and D). NaHS pre-treatment also significantly reduced effluent LDH during early, middle and late reperfusion (Fig. 2E). These results strongly indicate that NaHS pre-treatment protects against cardiac ischemia/reperfusion injury.

**Figure 3:**
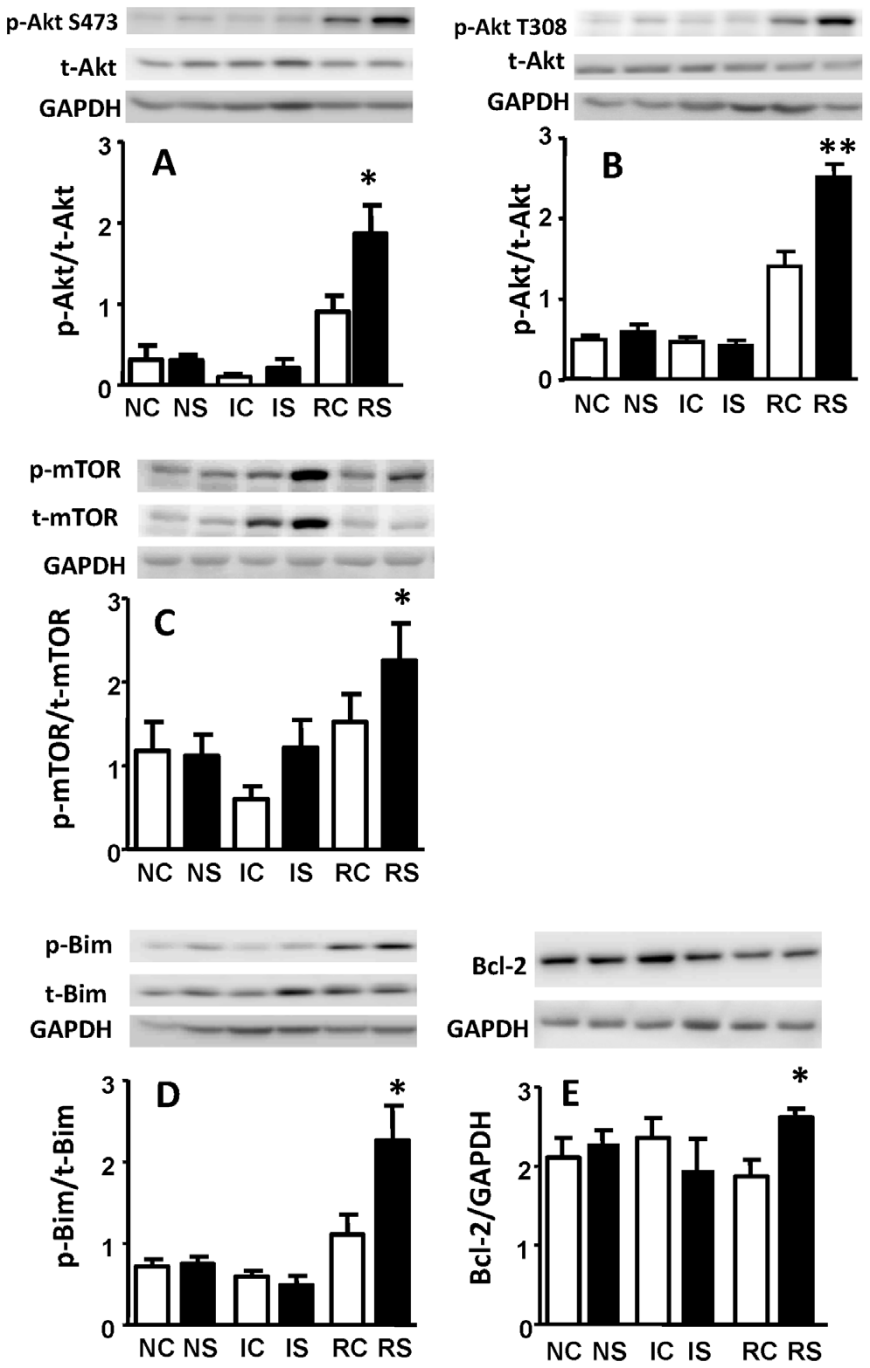
NaHS pretreatment regulates p-Akt, p-mTOR, p-Bim and Bcl-2 during normoxia, ischemia and reperfusion. Expression levels are presented in the ratio of (A) p-Akt Ser473 relative to total Akt, (B) p-Akt Thr308 relative to total Akt, (C) p-mTOR relative to total mTOR, (D) p-Bim relative to total Bim, (E) Bcl-2 relative to GAPDH. GAPDH was used as a protein loading control. NC = Normoxia Control, NS = Normoxia NaHS treatment, IC = Ischemia Control, IS = Ischemia NaHS pretreatment, RC = Reperfusion Control, RS = Reperfusion NaHS pretreatment. *P<0.05 vs. RC by two-tail unpaired t test.

To assess whether Akt activation occurs in hearts pre-treated with NaHS we freeze-clamped NC, IC, RC, NS, IS, and RS hearts, and measured their total Akt and phosphorylated Akt at Ser473 and Thr308 using Western blot analyses. We find that NaHS did not affect basal level Akt phosphorylation. Phosphorylations at both sites decreased during ischemia even with NaHS treatment. While reperfusion itself recovered Akt phosphorylation as previously reported [30], NaHS pre-treatment further doubled the phosphorylations of myocardial Akt during reperfusion (Fig. 3A and B).

Interestingly, increases of both phosphorylated and total mTOR also occurred in ischemic compared to control or reperfused hearts (Fig. 3C inset; IC vs. NC or RC). NaHS pretreatment dramatically enhanced these increases (Fig. 3C inset; IS vs. IC). Since the myocardial content of both forms of mTOR increased, no significant change in the phospho-to-total mTOR ratio occurred during ischemia (Fig. 3C, panel). Using GAPDH as an internal standard, however, phospho- and total mTOR increased about 2-and 3-folds respectively in ischemic compared to normoxically perfused hearts (Figure 3C; inset IC vs. NC). NaHS pretreatment further increased myocardial phospho- and total mTOR relative to GAPDH to about 5 fold (Fig. 3C inset; IS vs. NS). In control hearts, phospho- and total mTOR levels returned to normal during reperfusion (Fig. 3C panel & inset; RC vs. NC). However, in NaHS pretreated hearts, phospho-mTOR remained high during reperfusion (p<0.05; Fig. 3C panel & insets; RS vs. RC or NC). These data demonstrate that NaHS enhances mTOR phosphorylation in both ischemic and reperfused hearts.

### H_2_S up-regulates Bcl-2 pro-survival pathway

We further investigated whether Bcl-2 family members are downstream targets of Akt in the setting of H_2_S cardioprotection. In this study we examined Bcl-2, a cell survival promoter, and Bim, a cell death initiator. NaHS had no impact on the phosphorylation of Bim and Bcl-2 in normoxic and ischemic hearts but did increase both p-Bim and Bcl-2 significantly during reperfusion (Fig. 3D and E, respectively).

### mTORC2 phosphorylation of Akt contributes to H_2_S cardioprotection

**Figure 4:**
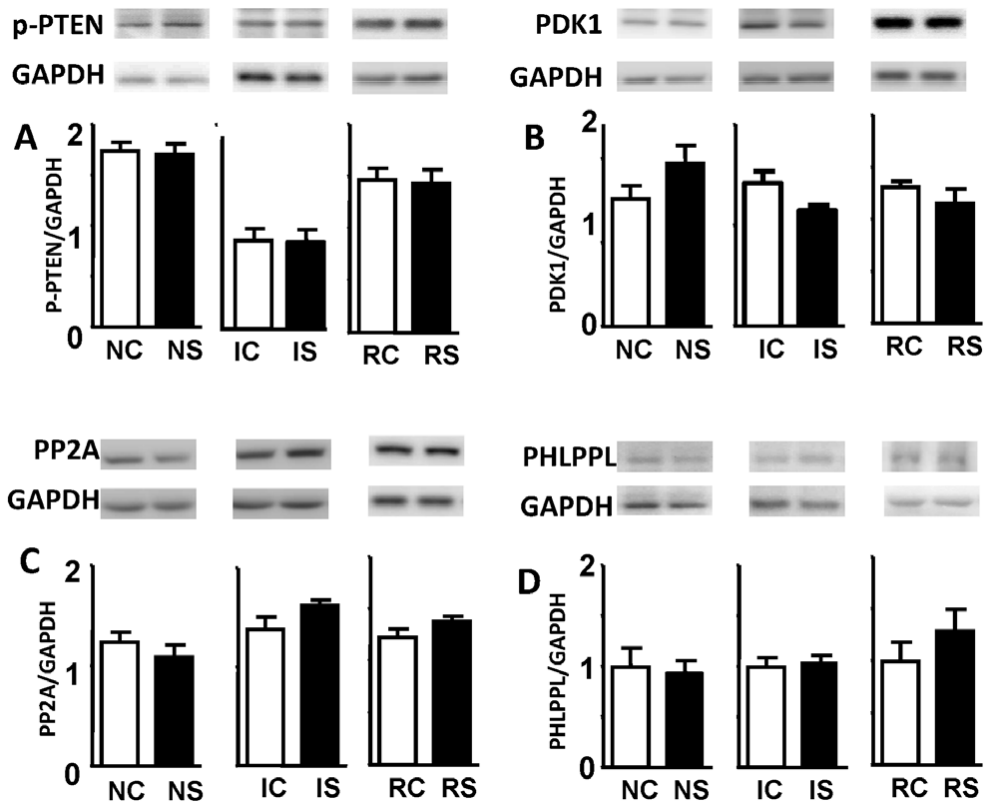
The effect of NaHS on the regulation of Akt kinase PDK1 and phosphotase PTEN, PP2A and PHLPPL. Expression levels were normalized with GAPDH. Expression level is presented in (A) p-PTEN, (B) PDK1, (C) PP2A and (D) PHLPPL. No significant differences between Reperfusion Control (RC) and Reperfusion NaHS-pretreatment (RS) were observed.

The phosphorylation states of Akt Ser473 and Thr308 regulate its activity. In order to better define the mechanism of Akt activation during H_2_S cardioprotection, we investigated whether the PDK1 and PTEN kinases and/or the PP2A and PHLPPL phosphatases contribute to H_2_S cardioprotection. We find that NaHS pretreatment does not affect PTEN, PDK1, PP2A or PHLPPL either during normoxia or ischemia and reperfusion (Fig. 4). mTORC1 is downstream target for Akt and mTORC2 can act as upstream kinase which phosphorylates Akt [31]. We thus tested whether the mTORC2 was the key regulators of Akt responsible for H_2_S cardioprotection.

**Figure 5:**
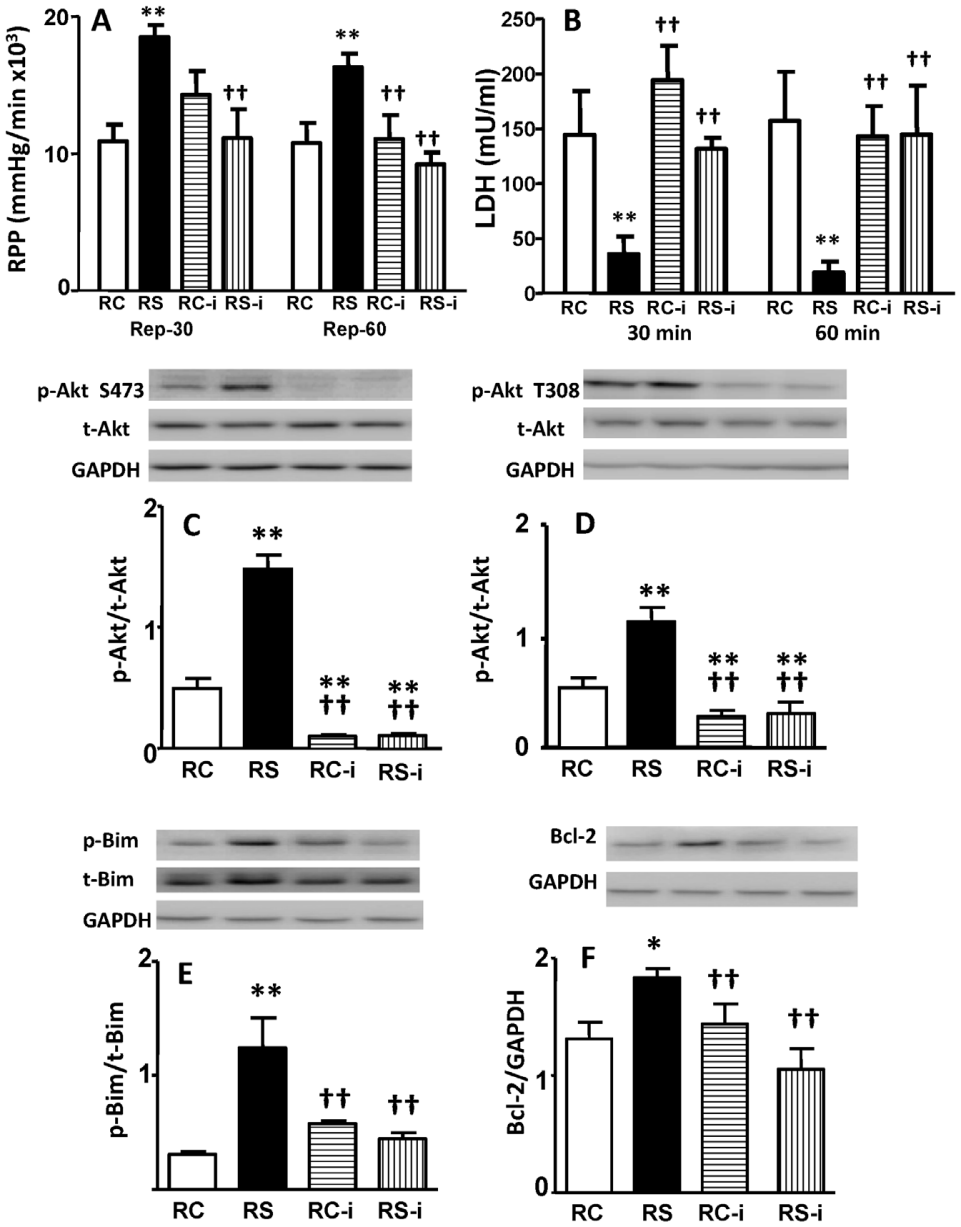
PP242, a specific mTOR inhibitor, abolishes NaHS pretreatment-induced cardiac functional recovery and activation of Akt, Bim and Bcl-2. (A) PRR recovery, (B) LDH release at the 30 and 60 min of reperfusion, (C) ratio of p-Akt Ser473 relative to total Akt, (D) ratio of p-Akt Thr308 relative to total Akt, (E) ratio of p-Bim relative to total Bim, and (F) ratio of Bcl-2 relative to GAPDH. RC = Reperfusion Control, RS = Reperfusion NaHS pretreatment, RC-i and RS-i = Reperfusion Control and Reperfusion NaHS pretreatment, respectively, with 0.3 µM PP242. *p<0.05, **p<0.01 vs. RC, ^††^p<0.01 vs. RS by one-way ANOVA followed by Bonferroni post-hoc analysis.

To investigate whether mTORC2 actives Akt in H_2_S cardioprotection we tested whether PP242, a specific pharmacological inhibitor of mTOR [32], affects heart function and changes Akt phosphorylation during reperfusion. Importantly, we find that 0.3 µM PP242 abolished H_2_S cardioprotection as indicated by loss of RPP recovery and increased LDH release at 30 and 60 min of reperfusion (Fig. 5A and B). In addition, this concentration of PP242 reduced p-Akt Ser473 and Thr308 compared to untreated or NaHS-pretreated hearts (Fig. 5C). It decreased the phosphorylation of Bim and abolished the enhanced level of Bcl-2 expression seen in NaHS treated reperfused hearts (Fig. 5E and F). It was not a surprise that PP242 also affected both p-Akt Ser473 and Thr308 in non-treated hearts, since there was an overshoot recovery of Akt phosphorylation in reperfusion compared to significant decrease in ischemia. However, it had no additional adverse effect on Bcl-2 and Bim. While PP242 may inhibit both mTOR Complex 1 and 2, these complexes have distinct roles in regulating Akt as knockout of mTORC2 reduces Akt phosphorylation, while mTORC1 knockout does not [33]. Therefore, these results support the proposition that mTORC2 mediates Akt phosphorylation in NaHS-treated hearts.

### NaHS increases the survival of ischemic H9c2 cells and PP242 prevents this change

**Figure 6:**
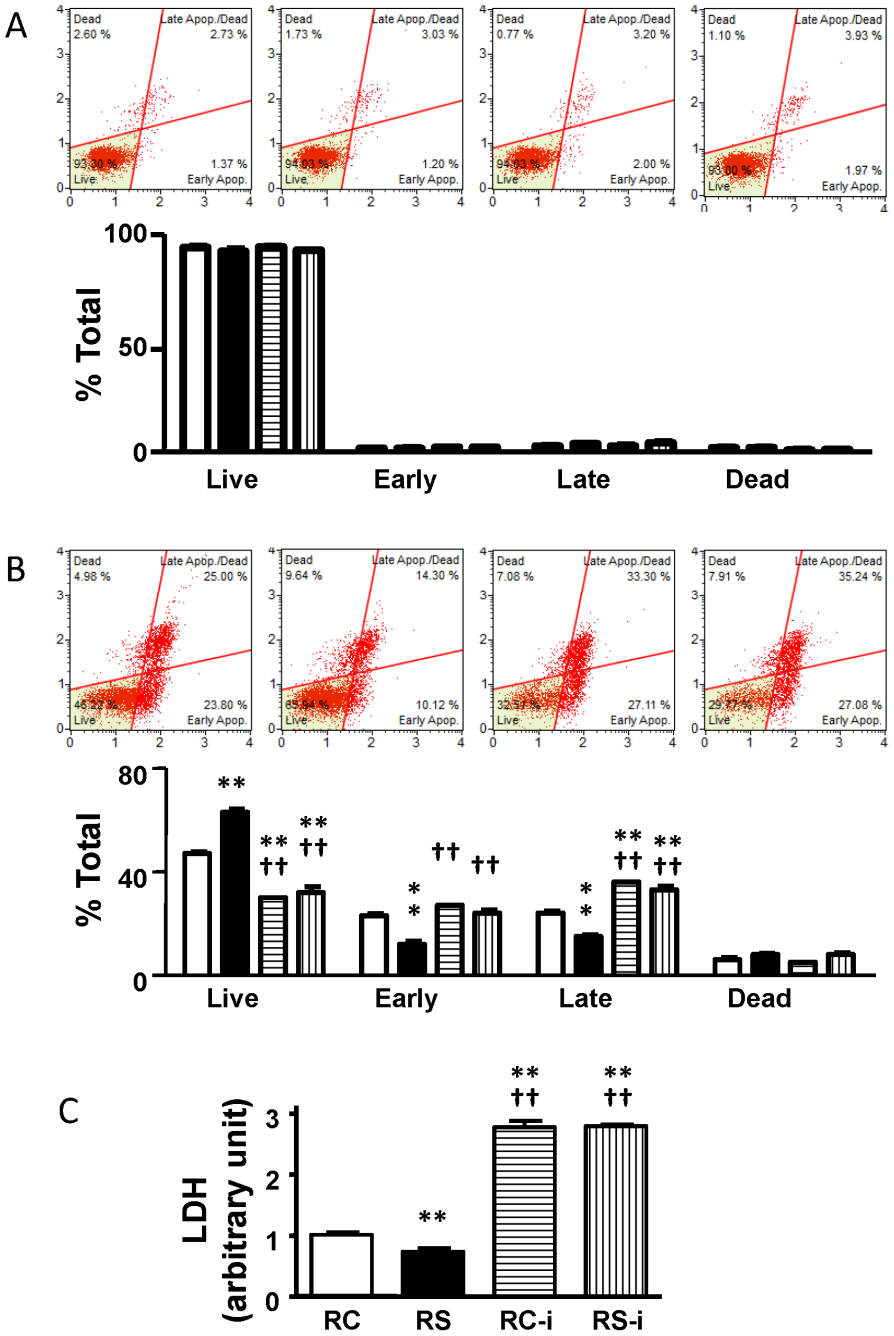
PP242, a specific mTOR inhibitor, blocks NaHS pretreatment induced anti-apoptotic effect in H9c2 cells. (A) and (B) Profiles of cell viability and apoptosis under normoxia and hypoxia conditions. (C) LDH release. RC = Reperfusion Control, RS = Reperfusion NaHS pretreatment, RC-i and RS-i = Reperfusion Control and Reperfusion NaHS pretreatment, respectively, with 0.3 µM PP242. **p<0.01 vs. RC, ^††^p<0.01 vs. RS by two-way (A and B) and one-way (C) ANOVA followed by Bonferroni post-hoc analysis. N = 3, each with 3 technical triplicates.

We performed additional studies testing whether H_2_S regulates Akt/Bcl-2 survival signaling so as to reduce apoptosis. H9c2 cells were cultured under normoxic conditions or underwent 15 hr of hypoxia followed by 2 hr of culturing under normal conditions. Neither NaHS nor PP242 induced apoptosis in normoxic cells (Fig. 6A). Under hypoxia conditions, however, NaHS significantly increased cell survival, reduced early and late apoptosis, and reduced LDH release (Fig. 6B and C). Importantly, PP242 reversed the anti-apoptotic effects which NaHS elicits.

### NaHS post-treatment protects hearts against ischemia/reperfusion through mTORC2-mediated activation of Akt pro-survival signaling pathway

**Figure 7:**
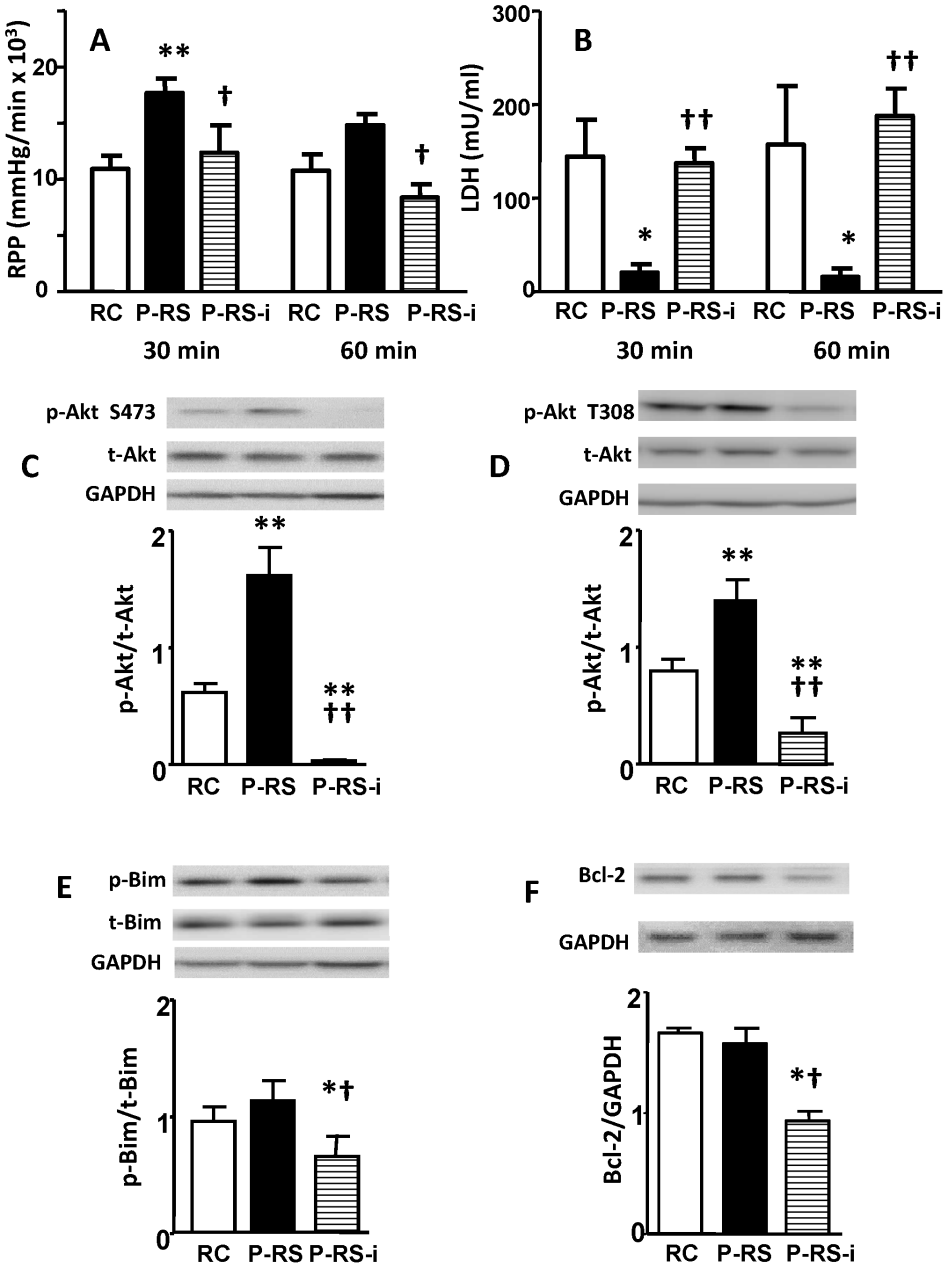
PP242, a specific mTOR inhibitor, abolishes NaHS post-treatment- induced cardiac functional recovery and activation of Akt, Bim and Bcl-2. (A) RPP recovery, (B) LDH release at the 30 and 60 min of reperfusion, (C) ratio of p-Akt Ser473 relative to total Akt, (D) ratio of p-Akt Thr308 relative to total Akt, (E) ratio of p-Bim relative to total Bim, and (F) ratio of Bcl-2 relative to GAPDH. RC = Reperfusion Control, P-RS = Reperfusion NaHS post-treatment, P-RS-i = Reperfusion NaHS post-treatment with 0.3 µM PP242. *p<0.05, **p<0.01 vs. RC, ^†^p<0.05, ^††^p<0.01 vs. P-RS by one-way ANOVA followed by Bonferroni post-hoc analysis.

Clinically effective cardioprotection would most likely occur only in the setting of acute myocardial reperfusion, not with pre-treatment. Hence we tested whether 50 µM NaHS could salvage heats if given only during reperfusion. In our preliminary study, with 10 min of NaHS post-treatment no significant protective effect could be detected (data not shown). In contrast, 30 min of NaHS post-treatment starting at the beginning of reperfusion, improved cardiac recovery. This improvement gradually abated and function did not differ from control by 60 min reperfusion (Fig. 7A). LDH release remained low (Fig. 7B) at 30 and 60 min reperfusion. Similar to pretreatment results, these beneficial effects were completely blocked by PP242. NaHS post-treatment up-regulated Akt phosphorylation at Ser473 and Thr308 as well and this effect was also abolished by PP242 (Fig. 7C and D). However, NaHS post-treatment failed to affect Bim phosphorylation and Bcl-2 levels. With PP242, Akt phosphorylation was reduced to almost undetectable levels, whilst p-Bim and Bcl-2 were down-regulated to significantly lower levels compared with control and NaHS post-treatment groups (Fig. 7E and F).

## Discussion

We investigated the molecular mechanism through which the H_2_S donor NaHS increases myocardial Akt phosphorylation to provide cardioprotection in an isolated heart model of ischemia/reperfusion. We demonstrated that PP242, a specific mTOR inhibitor, blocks this cardioprotection. Since mTORC2 and mTORC1 have clearly distinguished roles as an Akt activator and Akt downstream target, respectively, our data suggest that mTORC2 activates Akt in this model of cardioprotection. We further confirmed that Akt downstream effectors may include Bim (Ser 69) and Bcl-2, which can protect mitochondrial membrane stability and inhibit apoptosis following ischemia/reperfusion. The anti-apoptotic effects of H_2_S were confirmed in cultured H9c2 cells. Given at reperfusion, NaHS has weaker protective effects via a similar mechanism.

We chose NaHS as the most suitable source of H_2_S for isolated heart studies since NaHS releases H_2_S rapidly and the concentration of H_2_S present in the perfusate can be precisely controlled. Specifically, 50 µM NaHS dissolved in the perfusate generates ∼10 µM of H_2_S [26]. The higher concentration of 100 µM NaHS was used previously [7] but our preliminary study showed that this dose caused vasodilatation and reduced heart function whereas 50 µM NaHS did not. Thus we believe this lower dose is suitable for our purpose.

Akt or protein kinase B is a serine/threonine kinase and the three isoforms of which are all activated by phosphorylation at serine and threonine residues; Ser473/Thr308 in Akt1, Ser474/Thr309 in Akt2, and Ser472/Thr305 in Akt3 [34]. Heart contains high levels of Akt1 and Akt2, and Akt1 primarily regulates cell survival signaling whilst Akt2 is important in insulin signaling [35]. In this study we specifically investigated Akt1 activation using p-Akt Ser473 and Thr308 specific antibodies. Phosphorylation at both Ser473 and Thr308 are required for full Akt1 activation. We evaluated cardiac Akt phosphorylation under a variety of experimental conditions, and our data confirm that the protective effect of H_2_S associates with increased Akt phosphorylations at Ser473 and Thr308 during reperfusion. H_2_S does not affect Akt phosphorylation during normoxia or ischemia.

We undertook a series of experiments to evaluate the molecular mechanism through which NaHS might activate Akt and provide cardioprotection. PI3K kinase generates a lipid second messenger phosphoinositide-3, 4, 5- tri-phosphate (PIP3) from phosphoinositide-3, 4- bisphosphate (PIP2). PIP3 recruits Akt to the membrane and once there, multiple kinases, including PDK1 and/or mTORC2 phosphorylate Akt. LY294002, a putative PI3K-specific inhibitor, abolishes H_2_S-induced Akt phosphorylation and cardioprotection [7], [15]. Therefore, it is accepted that PI3K is the likely mediator of H_2_S-induced Akt phosphorylation. Because of critical role of Akt in the regulation of cell survival, it is possible that there is a “fail-safe” mechanism involved in multiple kinases and phosphatases in the regulation of activity state of Akt. Indeed, multiple kinases and phosphatase regulate Akt activity. These alternate regulators have not yet been investigated in H_2_S cardioprotection. For example, the tumor suppressor PTEN, a lipid phosphatase, dephosphorylates PIP3 to PIP2 which decreases Akt activity [36]. Yet again, PDK1 can be directly activated by extracellular stimuli, or be phosphorylated by PIP3 [18], [37]. Activated PDK1 phosphorylates and activates Akt Ser308. The phosphatase PP2A dephosphorylates Akt thereby decreasing its activity [38]. The PHLPP family has two protein phosphatases, PHLPP and PHLPPL; both directly regulate Akt activity through dephosphorylation. In a lymphoma cell line, it is demonstrated that 60% reduction in expression of PHLPPL increases Akt-Ser473 several fold [22]. Our data indicate that none of PTEN, PDK1, PP2A or PHLPPL plays a role in H_2_S-induced Akt activation.

Given the fact that NaHS significantly increases Akt phosphorylation in reperfused hearts while the preceding four regulators of Akt activity do not change in this setting, we assessed whether mTOR might catalyze the increase in Akt phosphorylation. This possibility seems likely as the IC_50_ of LY294002 for mTOR and PI3K are 5 and 3 µM respectively [17] and 10–15 µm of LY294002 was used in the initial studies of PI3K/Akt in H_2_S cardioprotection [7], [15]. In addition, PI3K activation of Akt is a complex process, requiring binding of PIP3 to Akt, recruiting of Akt to membranes, and its subsequent phosphorylation by multiple kinases including mTORC2 [18]. Since mTORC1 is a downstream substrate of Akt and mTORC2 can phosphorylate Akt at Ser473 [19], we tested the hypothesis that the mTORC2 might be one alternative mechanism to induce Akt phosphorylation during H_2_S cardioprotection. We chose a pharmacological approach as currently available mTOR antibodies recognize only the core mTOR protein and, therefore, cannot differentiate between mTORC1 and 2. Thus we used PP242, a potent and specific mTOR inhibitor, to address whether mTORC2 plays a role in increasing Akt phosphorylation during H_2_S cardioprotection. While PP242 inhibits both mTORC1 and 2, the fact that Akt is not a substrate for mTORC1 [33] suggests that mTORC2 is the kinase which phosphorylates Akt in H_2_S-induced cardioprotection [32]. We found that 0.3 µM of PP242 completely abolished the increase in Akt Ser473 and Thr308 phosphorylation that occurs in reperfused hearts pre-treated with NaHS. Although mTORC2 does not directly phosphorylate Akt Thr308, it facilitates the phosphorylation of Akt Thr308 caused by PDK1 [33]. The activity of PDK1 was not regulated by NaHS. So it is reasonable to suspect that increased and decreased Akt Thr308 phosphorylation by NaHS and PP242, respectively, is secondary to Akt Ser473 in this study. Our data reinforce that mTORC2 regulated Akt Ser473 phosphorylation is a key mediator of H_2_S-induced cardioprotection. Importantly, this decrease in Akt phosphorylation had functional consequences with abrogation of mechanical cardioprotection. Furthermore, PP242 also completely abolished the effect of NaHS on the downstream Akt targets Bim and Bcl-2 (Fig. 5). These data lead us to conclude that mTORC2 may mediate, in part, H_2_S-induced Akt phosphorylation. However, since PP242 also inhibits mTORC1 additional studies are required to more firmly establish our conclusion and examine whether mTORC1 might be a potential downstream effecter. A recent study demonstrated that increases in mTORC2 and Akt Ser473 phosphorylation lead to cardioprotection against ischemic injury [39]. This study highly supports our finding and conclusion.

Interestingly, both mTOR phosphorylation and total mTOR increase in ischemia and reperfusion compared to control. Importantly, Akt phosphorylation significantly increases in reperfused hearts compared to normoxic and ischemic hearts. This intriguing but yet unexplained phenomenon, that is the increase of mTOR and downstream p70S6K phosphorylations and the increase of Akt and S6K phosphorylations, has been observed by two different groups respectively [30], [40]. It may be that mTOR activation occurs early during ischemia in advance of Akt phosphorylation. Such a sequence of events might be a necessary preparation for cell survival.

Proteins of the Bcl-2 family can either promote cell survival, e.g. Bcl-2, or initiate cell killing, e.g. Bim [41] in ischemia/reperfusion injury. The pro- or anti-apoptotic effect is mediated through controlling mitochondrial permeability [42]. Whether they play a role in H_2_S-induced cardiac protection has never been investigated. Phosphorylation of Bim at Ser69 in response to survival factors might lead to reduced apoptosis through releasing Bim/Bax interaction [43] and increasing Bim degradation [44]. On the other hand, increased expression of Bcl-2 leads to pro-survival effect by stabilizing mitochondrial membrane permeability [41]. Our results show that both Bcl-2 and p-Bim are upregulated. Future studies will test whether the Bax subgroup of Bcl-2 effectors of apoptosis also plays a role in H_2_S cardioprotection. Bax interacting with Bcl-2 or Bim respectively inhibits or facilitates the translocation of Bax to the mitochondrial outer member and attacks the mitochondrial permeability transition pore [45], [46]. Thus assessing the translocation of Bax to mitochondria could be a worthwhile extension of the current sets of data.

We have examined the effect of NaHS post-treatment on recovery of heart function during reperfusion. Our results indicated that NaHS post-treatment improves cardiac functional recovery and reduces LDH release. This protective effect, which is also mediated through Akt, can be abolished by PP242. Compared with pretreatment, a few differences should be mentioned: (1) in order to achieve significantly improved cardiac functional recovery, at least 30 min NaHS post-treatment was needed; (2) improved cardiac function was only observed at the 30 min of reperfusion but failed to be detected at the 60 min of reperfusion; (3) although Akt phosphorylation was upregulated, its downstream effectors p-Bim and Bcl-2 were not detectably increased; (4) administration of PP242 completely abolished all the beneficial effects, and significantly reduced p-Bim and Bcl-2. Taking these data together, we believe that post-treatment with NsHS induces similar though weaker protective effects via the same mechanisms as pretreatment. Further protective effect might be achieved by increasing tissue H_2_S concentration and/or by prolonged treatment time.

A limitation of this work is that a role of mTORC2 in the phosphorylation of Akt was not directly measured. A pharmacological inhibitor PP242 inhibits both mTORC2 and C1, therefore whether mTORC1 plays a role in H_2_S-induced cardioprotection was not elucidated. However, due to distinguished roles of mTORC2 and C1 as Akt upstream to phosphorylate Akt and downstream to be phosphorylated by Akt respectively [33], it is highly possible that H_2_S phosphorylates Akt through mTORC2. An mTORC2 rictor and C1 raptor knockdown or silencing study will demonstrate their roles in H2S-induced cardioprotection directly and clearly.

**Figure 8:**
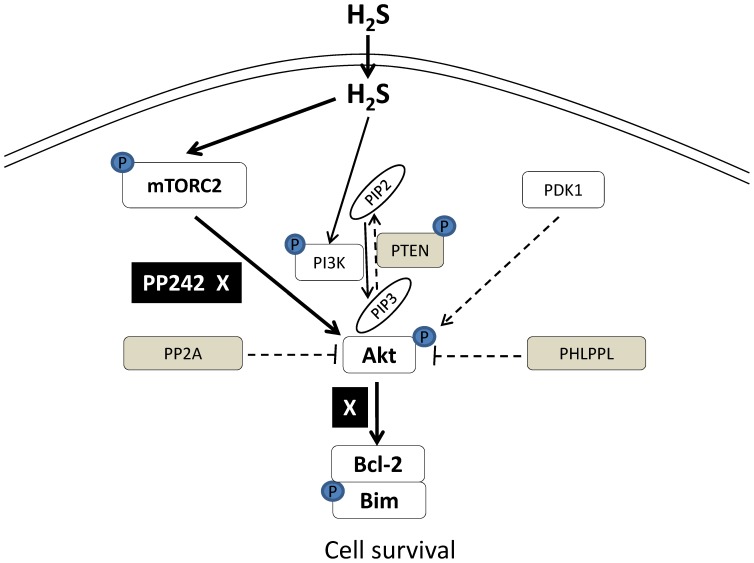
A schematic diagram illustrates the mechanisms of H_2_S-induced cardiac protection. In addition to PI3K regulation, H_2_S increases cell survival through mTORC2-mediated activation of Akt/Bim and Bcl-2 pro-survival cell signaling pathway. PI3K generates PIP3 from PIP2. PIP3 recruits Akt to the membrane and where Akt is phosphorylated by mTORC2. Solid lines indicate positive and dotted lines negative findings in H_2_S-induced cardioprotection. PP242 inhibits mTORC2 induced Akt phosphorylation, Bcl-2 expression and Bim phosphorylation.

In conclusion, our results confirmed the protective effects of H_2_S against cardiac I/R injury by both pre- and post-treatment with NaHS. As illustrated in Fig. 8, our data support the idea that mTORC2-mediated activation of Akt contributes to the cardioprotective effects of H_2_S in addition to the well accepted role of PI3K in this process. This alternative mechanism may allow for Akt to act as a ‘central sensor’ for myocyte survival or death. That is, multiple sensor kinases may reside upstream of Akt, and Akt may integrate multiple input signals from these kinases, including PI3K and mTORC2, to positively or negatively affect myocyte survival. Our data suggest that the cardioprotection of H_2_S is mediated through Akt downstream Bcl-2 and Bim, a pro-survival cell signaling pathway. Akt kinase PDK1 and phosphatases PTEN, PP2A and PHLPPL are not involved in H_2_S-induced Akt activation.
